# Infective Endocarditis in Patients With a History of Intravenous Drug Abuse

**DOI:** 10.7759/cureus.91270

**Published:** 2025-08-30

**Authors:** Marcin Bursy, Adam Dudek, Wojciech Szkudlarek, Jan Linkiewicz, Wojciech Lizurej, Aleksandra Buda, Marta Kubik, Aleksander Bogdanski, Kalina Bogdanska, Maksym Mazur

**Affiliations:** 1 General Practice, University Hospital in Poznań, Poznań, POL; 2 Hospital-Based Medicine, Medical Center HCP, Poznań, POL; 3 Medical School, Poznań University of Medical Sciences, Poznań, POL; 4 Hospital-Based Medicine, University Hospital in Poznań, Poznań, POL; 5 Hospital-Based Medicine, Poznań University of Medical Sciences, Poznań, POL; 6 Research, Poznań University of Medical Sciences, Poznań, POL; 7 Hospital-Based Medicine, Charité Universitätsmedizin Berlin, Berlin, DEU; 8 Medical Education, Wrocław Medical University, Wrocław, POL

**Keywords:** angiovac and infective enocarditis, echocardiography findings, right sided infective endocarditis, valvular endocarditis, drug abuse

## Abstract

Infective endocarditis (IE) in people who inject drugs (PWID) has emerged as a growing public health concern. It entails two main issues future medicine will have to face: an increase in patients with substance abuse and the evolution of new microbial threats. The analysis of characteristics of the disease in this group of patients enables us to draw differentiating points. IE in PWID predominantly involves the right side of the heart, mainly the tricuspid valve. The prevalence of the disease is high in developed countries, in regions with a strong impact of drug abuse, particularly in the younger part of populations facing socioeconomic difficulties linked to changing life conditions in postindustrial economic systems. Staphylococcus aureus is by far the most frequent pathogen responsible for the infection in these patients, proportionally outnumbering the prevalence of this species in non-drug users. Also, higher rates of Gram-negative and fungal infections are posing a serious challenge in the treatment process.

The Duke Criteria remain the common diagnostic methodology in all cases of IE, with echocardiography - both transthoracic echocardiography (TTE) and transoesophageal echocardiography (TEE) - being staples for imaging. The identification of the pathogen includes both standard microbiologic blood culture tests and the use of advanced biomolecular methods. Management in an optimal therapeutic process requires targeted antimicrobial therapy, which in many cases has to be supported by invasive surgical or percutaneous interventions. The widening spectrum of surgical procedures, such as percutaneous vegetation debulking, transcatheter valve-in-valve implantation, or minimally invasive cardiac surgery, represents a positive change for patients in poor clinical condition who are not suitable for standard cardiothoracic procedures.. While short-term survival seems promising at first, the long-term results are still dissatisfying. The main area for improvement is the need for a multidisciplinary approach that integrates infection management and prevention with addiction treatment. An innovative approach combining these factors could lead to a better prognosis in PWID with IE in the future.

## Introduction and background

Infective endocarditis (IE) is an infectious disease of the endocardium, predominantly affecting the valves. Historically, it has been associated with structural abnormalities of the valves, prosthetic devices, or rheumatic heart disease. In the past, patients diagnosed with endocarditis were usually older adults with several comorbidities. However, a new factor has emerged as significant in the epidemiology of endocarditis in the past two decades. It involves the rising incidence of intravenous drug use (IVDU), particularly involving opioids [1]. Hence, IE now presents more frequently in younger, previously healthy individuals who inject drugs. The social background of the patients varies and cannot be linked explicitly to poverty, as the highest usage trends were noticed in the wealthy countries in Europe, the United States, and the Arabian Peninsula [1]. This shift poses unique challenges for diagnosis, treatment, and prognosis. As patients with a history of drug abuse tend to develop right-sided IE more often than other groups, they also present distinct clinical features such as septic pulmonary emboli and carry a different prognosis compared to non-drug-using endocarditis patients [2,3]. Furthermore, recurring bacteremia due to ongoing drug use, socioeconomic instability, and a lack of compliance necessitate an open-minded approach towards the patient while considering standard treatment pathways and surgical decisions. In this review, we examine the epidemiological, pathological, and clinical characteristics, diagnostic approaches, therapeutic management, and long-term prognosis of IE patients with a history of IVDU.

## Review

Epidemiology

The incidence rate of IE fluctuates from three to 10 individuals per 100,000 annually, with a varying but relatively high mortality rate [4]. It is essential to highlight the increasing number of cases presenting IE as a growing social concern. A report on cohort groups in several countries suggests that, after years of rising incident rates, the numbers of new cases have been reversed or at least plateaued within the second decade of the 21st century, with a further increase in recent years [5]. On the other hand, some studies report that the number of intravenous drug users in patients with endocarditis surpassed the number of patients who are not abusing narcotic substances [6]. A study focused on the Persian Gulf region draws the conclusion linking the higher incidence rates not only to the abuse of drugs in the local populations as a negative side effect of economic development, but also to the existence of important drug trade routes in the region. Further analysis of drug abuse patterns in this region reveals certain backgrounds of users, as both poor educational results and hailing from a high-income household have been identified as risk factors of drug use [1].

The epidemiological data suggest certain differences between patients with a history of abusing intravenous narcotics in comparison with the broader epidemiological spectrum. The right side of the heart, especially the tricuspid valve, located between the right atrium and the right ventricle, happens to be statistically more likely to be affected in drug-using groups [7]. This has to be linked with the fact of intravenous insertion of pathogens by people who inject drugs (PWID). These pathogens migrate through the bloodstream into the right part of the heart through the venous system. In a study performed in a large medical center, almost 60% of the time, the valves of the right heart were covered by the infective process, with the left side of the heart making up below 30% of all the cases. In about one in eight patients, both sides of the heart had been infected [2]. On average, the IVDU patients with IE are also younger and tend to be around 40 years old with a slight male predominance [8,9]. Common factors among IVDU patients include homelessness, mental health disorders, and legal problems [10]. These are also risk factors linked to therapy non-compliance, hospital readmission, and a negative long-term prognosis.

Another aspect of the difficulty in the management of IVDU patients with endocarditis is their low socioeconomic status and lack of access to healthcare in certain regions, since many affected persons live in rural areas. A study from the U.S. state of South Carolina also emphasized the lack of physicians specialized in treating and diagnosing infectious diseases in these areas. Patients who do not receive consultations with a specialist physician have poorer outcomes and are at higher risk of facing severe complications [11]. The comorbidities appearing most frequently in this group are HIV and chronic liver disease [12]. In a report published in 2022, over one-third of all patients were HIV-positive. This diagnosis is connected to a worse prognosis due to the immunodeficiency caused by the virus, which increases the risk of coinfection, reinfection, and developing AIDS-related comorbidities, carrying a poor prognosis. Patients from this group, in general, have a longer history of using drugs than others. [12]. The opioid epidemic is a catalyst and cofactor of growing incidence rates of IE in PWID, as heroin is the most often used narcotic substance. However, the spectrum of narcotics used by patients with IE is very broad and includes substances with diverse mechanisms of action and biochemical effects on the human body, such as cocaine, which is responsible for sympathetic stimulation [10,13].

Pathogenesis and microbiology

The final clinical presentation of IE initially involves a series of events within the damaged endothelium of the heart, typically the surface of the heart valves. This is followed by bacterial adhesion and an inflammatory reaction within the area of the damaged endocardium. While the cause in non-IVDU patients is related to turbulent blood flow or a preexisting defect of the valve, prosthetic valves, or intracardiac devices, drug users insert the microorganisms directly through the use of impure and contaminated materials during intravenous drug use [7,14]. The most common species responsible for IE is Staphylococcus aureus, estimated to be the pathogen in 60-70% of all cases in IVDU patients compared to less than one-third in non-drug users [7]. All species from the Streptococcus spp. and Streptococcus spp. add up to be the responsible pathogen of approximately 80% of all cases [13]. There is a noticeable, rising prevalence of multidrug-resistant Staphylococcus aureus, including the methicillin-resistant strain (MRSA) [15]. The third most prevalent genus is the Enterococcus, which also has a significantly increased drug-resistance profile. 

An interesting gathering of pathogens is the HACEK group, consisting of Haemophilus spp., Aggregatibacter spp., Cardiobacterium spp., Eikenella corrodens, and Kingella spp. Pathogens forming this group are difficult to isolate in diagnostic blood cultures [15]. Gram-negative aerobic bacteria and Candida albicans cause IE through bloodstream infections and maintain a significant mortality rate. In a study by Tan et al., focusing on bloodstream infections right right-sided endocarditis has been associated with a lower mortality rate [16]. Bloodstream infections should be considered more likely in patients with IVDU, but also in patients with a history of intravenous catheterization during hospital stays. Gram-negative bacteria, for example, Pseudomonas spp., Serratia spp., and polymicrobial or fungal agents (Candida albicans) are more common in right-sided IE [17]. Fungal infections usually affect the endocardium in multiple locations or the tricuspid valve; however, an isolated infection within the pulmonary valve can also occur [18]. About 5% of all cases of endocarditis are blood culture-negative [14].

Clinical characteristics

The clinical presentation of IE can vary depending on the location of the inflammatory process within the heart, the type of pathogen, or existing comorbidities. In intravenous drug users, where right-sided IE is more common, symptoms may result from emboli in the pulmonary circulation as well as in the systemic circulation, leading to the development of respiratory manifestations [19]. Septic pulmonary emboli can be diagnosed in these patients even in the absence of clearly visible valvular vegetations on imaging tests. Unlike classical pulmonary embolism, which is caused by thrombotic material, septic emboli in IE originate from infected valvular vegetations and spread to the pulmonary circulation [3]. Symptoms frequently reported by patients during anamnesis include: pleuritic chest pain, cough, hemoptysis, shortness of breath, yet they are not present in most cases, even in patients with a pulmonary embolism as a complication of IE [7]. These symptoms require clinicians to include various pulmonary diseases such as bacterial pneumonia, lung abscess, tuberculosis, pulmonary embolism, or even malignancies in their differential diagnosis.

Several constitutional symptoms, including fever, night sweats, and malaise, are non-specific but common in patients with IE in both sides of the heart [7]. Peripheral stigmata, for example, Janeway lesions, Osler nodes, and splinter hemorrhages, are a rare presentation of the disease [13]. Physical examination results are also dependent on the valves involved; particularly, heart murmurs in the auscultation areas can be a clue for which valves or side of the heart are involved. The murmurs usually appear in the systolic phase, as a result of regurgitation in the atrioventricular valves [1,20]. In some cases, despite a significant haemodynamic disturbance, murmurs might not be present. Laboratory findings comprise elevated inflammatory markers C-reactive protein (CRP), erythrocyte sedimentation rate (ESR), leukocytosis, or normocytic anemia [21]. Complications at admission present in IVDU patients include renal failure, cardiogenic shock, or multiple septic emboli. Early recognition is critical to the application of the necessary treatment.

Diagnostics

Early diagnosis of IE is crucial as it reduces morbidity, complications, and progression of both the main disease and comorbidities. In every epidemiological group of patients with IE, the Duke Criteria remain the main framework for establishing the proper diagnosis in both right- and left-sided endocarditis [1]. It integrates tests from different fields such as imaging, microbiology, and laboratory tests, as well as clinical findings. The diagnostic criteria are partitioned into major and minor criteria. The major criteria group includes findings in echocardiographic imaging and positive results for blood cultures within groups of IE-typical microorganisms [22]. The minimum amount of three sets should be drawn from different sites with a time interval of 6h [7]. It is crucial to take the blood samples before the first dose of antibiotics is administered [7]. Transthoracic echocardiography (TTE) is the first-choice imaging tool and is sufficient in most cases to obtain a result that matches the Duke Criteria. Although transoesophageal echocardiography (TEE) is proven to have a higher sensitivity, especially in patients with prosthetic valves, abscesses, or leaflet perforation [7,23]. TTE is also an important tool for assessment of the severity of tricuspid regurgitation in right-sided IE [24].

Other imaging modalities include PET/CT(positron emission tomography) with the use of 18F-FDG (fludeoxyglucose); although they are rarely applied, they manage to constitute a useful diagnostic instrument [13]. Moreover, CT, MRI, and SPECT/CT (single photon emission CT) have also been proven as helpful imaging techniques [15]. These methods can also be used as a way of confirming a hypothetical diagnosis in cases where the characteristics of previous findings remain doubtful [25]. Molecular diagnostics like PCR or rRNA sequencing can be used, which seems to be a valuable tool in cases of negative blood culture results or cases with a polymicrobial profile [23]. New imaging methods and molecular diagnostics have been included in the 2023 revised Duke Criteria [22]. For a definitive diagnosis of IE, the following criteria must be fulfilled: two major criteria, one major criterion, and three minor criteria or five minor criteria [26]. The differential diagnosis requires a cautious approach with cardiological (myxoma) and rheumatological (e.g., SLE, Antiphospholipid syndrome) diseases being the most important to differentiate IE with [7].

Non-invasive treatment

There are two equally important aspects regarding the treatment of a patient with IE [7]. The infection itself through antibiotics and mitigating the systemic consequences of the infection and the morphological, physiological abnormalities it has caused. Further concerns in IVDU patients include long-term outcomes, affected by a variety of factors, including socioeconomic status or access to medical care. Empiric antibiotic therapy should be initiated after taking blood culture samples for microbiologic testing. While deciding which agents should be used during empirical antibiotic therapy, information and data involving the Gram stain of the pathogen should be brought up [26]. The duration of pharmacological therapy varies but should last at least four weeks. It is strongly recommended that the shaping of a specific treatment plan should be preceded by a consultation with a specialist in infectious diseases and according to regional guidelines [26]. Guidelines for treatment can be adjusted to the epidemiological data of the region, focusing on the common pathogens in each region and possible pharmacological resistance profiles of these microorganisms. Usually, the antibiotic agents include penicillin, ceftriaxone, and a representative of the aminoglycoside group [27]. In Staphylococcal infections with high risks of MRSA strains, vancomycin is the antibiotic of choice [28].

Most of the IVDU patients are given a full intravenous treatment, with a lower proportion of patients undergoing a conversion from intravenous to oral antibiotic treatment. However, there are numerous types of treatment. One of them, outpatient parenteral antibiotic therapy (OPAT), is met with scepticism in PWID, as only a minority of surveyed facilities offered this treatment modality [29]. One of the reasons for distrust towards OPAT could be the risk of misuse and lack of adherence in this patient group. Fungal infections mainly caused by Candida albicans require intensive antifungal treatment with the use of fluconazole, amphotericin B [18]. Collis et al. have shown the benefits of a multidisciplinary approach in patients with isolated tricuspid valve endocarditis [30]. The non-invasive treatment part of therapy does not have a specific endpoint. After discharge from the hospital, a multidisciplinary treatment plan involving many paths is a road to recovery and readmission prevention. This includes, among others, cardiological, psychiatrical, psychological, and social care [26]. A typical complication in PWID is withdrawal syndrome, particularly opioid withdrawal. The best way to manage this condition is the substitution of specific agents from the opioid group, like buprenorphine or methadone. Although they bind to the same receptor as other opioids used by PWID, the pharmacodynamic profile of these medications makes it possible to mitigate the consequences of opioid abstinence in PWID [27].

Surgical treatment

Invasive treatment in IE is a viable option in a significant proportion of cases and should be considered. Approximately one-half of all IVDU patients are undergoing an operation in their course of treatment [31]. A higher proportion of surgeries in PWID are emergency surgeries, which can be linked to the fact that more patients from this group are admitted in a critical state in the perioperative period [31]. These patients also have a higher risk of reoperation, mortality rate, and poorer long-term outcomes. In right-sided endocarditis, surgical outcomes are better in patients with only the right side affected. Concomitant with the left side of the heart, for example, the mitral valve results in a worse outcome prognosis [32]. While looking at operative studies focusing on the tricuspid valve, slightly more than half of the patients required an invasive procedure. A majority of the patients receiving an invasive treatment had undergone a percutaneous aspiration instead of a regular cardiac surgery [33]. In a study by el Sabbagh et al., mortality was connected to vegetation size of over 2 cm.

Future directions include the increasing role of minimally invasive procedures like the percutaneous aspiration of valve vegetations [34]. In this study, the vegetations were successfully removed in three-fourths of patients in the sample. It is stated that the main goal of the aspiration of the vegetation is a significant reduction in the size of the lesion; the reasoning is that an increase in vegetation size contributes to a higher risk and severity of cardiac damage. The procedure is usually guided by both echocardiographic (TEE) and fluoroscopic vision. Before performing the procedure, it is important to select the appropriate device using the operator's clinical experience [34]. In this process, both features of the vegetation and characteristics of the patient should be taken into account. Aspiration techniques also have a key role in the treatment algorithm as they can be performed on patients who have been disqualified to undergo cardiac surgery [35]. The AngioVac, a system used for percutaneous removal of emboli or vegetations, applied in these procedures, was a useful strategy of intervention in patients with existing comorbidities, including cardiac dysfunction that often resulted in hemodynamic instability in the perioperative period [35]. A notable finding is that almost 90% of the patients had no complications after the procedure.

Prognosis

Long-term outcomes in IVDU patients can be challenging and depend on many variables that go beyond the boundaries of medical science. This hypothesis is based on data that suggests that short-term survival rates are high and do not differ from those in non-drug-using populations with IE [36]. However, the overall life expectancy is far worse when compared to the general population. This fact is linked to the risk of recurrent endocarditis, infectious diseases in other compartments of the organism, such as the central nervous system, or overdose. The point on the treatment timeline where mortality is high is the first hospital admission with drug-related IEC [37]. It is also stated that cardiac surgery does not improve the overall life expectancy in IVDU patients [36]. Survival rates also depend on the pathogen being the cause of the infection. Fungal and polymicrobial infections tend to be associated with a higher risk of mortality [7]. A study emphasizing the outcome in patients with severe tricuspid IE with acute tricuspid regurgitation with coexisting complications such as septic shock and multiple emboli showed that pharmacological and surgical treatment is sufficient to achieve a high five-year survival rate, although the perioperative mortality in patients who were qualified for surgery was almost 20% [24].

The future incidence rate of IE in PWID will be strongly linked to the number of drug users [38]. A segment of the readmissions in PWID is classified as preventable readmissions, their cause being recurrent substance abuse. The estimated costs of such preventable readmissions are very high [38]. Initial assessment and distinguishing between relapse and reinfection could benefit the diagnostic process. Relapse, meaning a recurrent infection with the same pathogen within six months of the initial diagnosis, is linked to unsuccessful therapy, while a reinfection is probably connected to continuous exposure to risk factors after treatment. From a social perspective, implementing policies and plans to prevent drug addiction in society and treating people with already existing addiction seem to be crucial methods for the primary prevention of endocarditis [39].

Larger and more formalized strategies are more likely to succeed as standardization of treatment methods reduces the risk of stigmatization and discrimination of patients. This cannot be achieved without gaining a deep understanding of the process of IVDU and addiction development. The care following the hospital discharge is likely to succeed in the long term if it prioritizes certain factors, such as focusing on the patient, and emphasizes multidisciplinary care without an overly extensive focus on one of its components [39]. A tentative option to lower the infection rates could be the facilitation of supervised injection sites, as IC in PWID is often caused by contaminated needles and other injection-related events [38]. Survival rates can be increased with the optimization of treatment strategies [5].

## Conclusions

IE among patients with a history of IVDU is a very complex clinical syndrome with significant distinctions compared to the general population. The differences are not only epidemiological or statistical, but pertain most importantly to the difficult process of treating IVDU patients. It is important to underline the need for multidisciplinary management comprising antimicrobial therapy, possible surgical intervention, addiction treatment, and care after discharge from the hospital. Developing appropriate guidelines or even basic strategies for this group could help improve the long-term survival rates. The aspects that require attention primarily include addiction treatment and lowering the risk of a reinfection. There is certainly scope for creating a formalized approach towards this group of patients in the form of a dedicated framework that would comprehend the complexity of the disease and the need for long-term commitment. The way to address this issue is not only medical and should not be discussed in a vacuum without taking into account social aspects such as housing or social work. Renewed attention should be paid to the fight against stigmatization and discrimination against PWID, ensuring that equitable care will be provided to them. This could lower the risks of relapse or further social exclusion of the patients.
